# A Plasmid Set for Efficient Bacterial Artificial Chromosome (BAC) Transgenesis in Zebrafish

**DOI:** 10.1534/g3.115.026344

**Published:** 2016-01-26

**Authors:** Fernando Fuentes, Eric Reynolds, Stephen W. Lewellis, Gayatri Venkiteswaran, Holger Knaut

**Affiliations:** * Developmental Genetics Program, Skirball Institute of Biomolecular Medicine, New York University Langone Medical Center, New York 10016; † Kimmel Center for Stem Cell Biology, New York University Langone Medical Center, New York 10016

**Keywords:** zebrafish, BAC transgenesis, gene expression

## Abstract

Transgenesis of large DNA constructs is essential for gene function analysis. Recently, Tol2 transposase-mediated transgenesis has emerged as a powerful tool to insert bacterial artificial chromosome (BAC) DNA constructs into the genome of zebrafish. For efficient transgenesis, the genomic DNA piece in the BAC construct needs to be flanked by Tol2 transposon sites, and the constructs should contain a transgenesis marker for easy identification of transgenic animals. We report a set of plasmids that contain targeting cassettes that allow the insertion of Tol2 sites and different transgenesis markers into BACs. Using BACs containing these targeting cassettes, we show that transgenesis is as efficient as iTol2, that preselecting for expression of the transgenesis marker increases the transgenesis rate, and that BAC transgenics faithfully recapitulate the endogenous gene expression patterns and allow for the estimation of the endogenous gene expression levels.

Transgenic animal models are essential experimental tools for unraveling gene function in normal development and disease. An ideal transgene recapitulates the endogenous gene expression pattern and levels. To achieve this, it must contain all the regulatory sequences in the gene of interest. Since these regulatory sequences are often far apart, an ideal transgenic construct frequently needs to span more than 100 kb of DNA. Large fragments (50–200 kb) of the genomes of most animal model systems have been cloned into bacterial artificial chromosomes (BACs). These BACs can be modified through recombineering (recombinant genetic engineering) to generate the transgenic construct of choice (Narayanan and Chen 2011).

The recent development of Tol2 transposase-mediated BAC transgenesis allows for the efficient integration of large DNA constructs into the zebrafish genome. This method uses minimal *Tol2* *cis*-sequences (iTol2) that are inserted into the BAC construct to flank the genomic DNA piece. The BAC construct with iTol2 is then coinjected with *Tol2* mRNA into one-cell-stage embryos. This approach increases the insertion rate of BAC transgenes from 0.5–5% (Nechiporuk *et al.* 2007; McGraw *et al.* 2008; DeLaurier *et al.* 2010) to around 20% (Suster *et al.* 2009a, 2009b, 2011; Bussmann and Schulte-Merker 2011).

Although this constitutes an impressive four- to 40-fold increase in the rate of transgenesis, this method relies heavily on the identification of BAC transgenic fish based on fluorescent proteins driven from the promoter of choice. This leads to the identification of BAC transgenic lines with sufficiently high expression levels for detection by eye, which likely results in a bias for overexpressing BAC transgenic lines—a problem if one wishes to study the protein of interest at endogenous gene expression levels. Also, promoters and insertions sites with fluorescent protein expression levels below the detection limit of the eye cannot be easily identified. To circumvent these problems, we developed a set of plasmids that harbor targeting cassettes for different BAC backbones and contain *Tol2* *cis*-sequences and fluorescent transgenesis markers. Using these targeting cassettes, we generated more than 70 BAC transgenic lines. We find that the BAC transgenesis rate is comparable to the iTol2 approach, but allows for the unbiased identification of BAC transgenic lines, and that presorting for expression of the transgenesis marker increases the transgenesis rate. We also find that BAC transgenics faithfully recapitulate the endogenous gene expression patterns, and allow for estimation of the endogenous gene expression levels if generated in an unbiased manner.

## Materials and Methods

### Construction of BAC backbone targeting cassette plasmids

For pTARBAC2-backbone-based BACs, targeting cassettes containing Tol2 sites (exon 4 and exon 1) flanking a bacterial selection cassette (FRT-galK-FRT, kanaR or tetR) followed by the lens-specific *cryaa* promoter (Kurita *et al.* 2003) driving a fluorescent protein (dsRed, superfolder GFP, Citrine or Cerulean), flanked by arms of homology to the *sacB* gene in pTARBAC2, were assembled in pBluescript vectors using PCR, restriction enzyme cloning, and Gibson cloning (Pédelacq *et al.* 2005; Gibson *et al.* 2009; Amiram *et al.* 2015). The 5′ and 3′ arms of homology are 231 bp and 242 bp fragments corresponding to nucleotides 2777–3007 and 3053–3294 of the pTARBAC2 backbone, respectively. Upon homologous recombination, these arms replace nucleotides 3008–3052 of the pTARBAC2 backbone, and disrupt the *sacB* coding sequence but do not interfere with propagation of the BAC. The targeting cassettes for the pTARBAC2 (around 4900 bp depending on the fluorescent protein and selection marker) were amplified using the following two primers: ggcggccgccaggcctaccca (forward) and taaagacacggcccgcgtttt (reverse).

For pIndigoBAC-536-backbone-based BACs, targeting cassettes containing Tol2 sites (exon 4 and exon 1) flanking a bacterial selection cassette (FRT-galK-FRT or tetR) followed by the lens-specific *cryaa* promoter driving dsRed, flanked by arms of homology to the lacZ gene in pIndigoBAC-536, were assembled in pBluescript vectors using PCR, restriction enzyme cloning, and Gibson cloning. The arms of homology were 320 bp fragments corresponding to nucleotides 409–728 and 761–1080 of the pIndigo-356 backbone, respectively. Upon homologous recombination, these arms replace nucleotides 409–728 and 761–1080 of the pIndigo-356 backbone, respectively, and disrupt the lacZ gene but do not interfere with propagation of the BAC. The targeting cassettes for pIndigoBAC-536 (around 5100 bp depending on the fluorescent protein and selection marker) were amplified using the following two primers: taaatagcttggcgtaatca (forward) and gtttctacacatatattcgc (reverse).

The pBluescript plasmids containing the BAC backbone targeting cassettes and their full DNA sequence are available from Addgene (http://www.addgene.org/).

### Construction of the BAC transgenes

For the *sdf1a:sdf1a-3xFlag-4xHA* BAC transgene, the BAC clone CH73-199F2 was modified in two ways by recombineering. First, the Tol2 sites and the *cryaa:dsRed* transgenesis marker were inserted into the BAC backbone. Second, the *3xFlag-4xHA* coding sequence was inserted between the last amino acid and the stop codon of *sdf1a* using seamless galK-mediated recombineering (Warming *et al.* 2005). Approximately 50 bp of homology upstream and downstream of the *sdf1a* stop codon were added to the *3xFlag-4xHA* cassettes by PCR. The final BAC transgene was characterized by *Spe*I and *Eco*RI restriction digestion, sequencing of PCR amplicons of the modified locus, and BAC-end sequencing.

For the *cxcr4b:Lifeact-Citrine* transgene, the BAC clone DKEY-169F10 was modified in two ways by recombineering. First, the Tol2 sites and the *cryaa:dsRed* transgenesis marker were inserted into the BAC backbone. Second, a cassette consisting of *Lifeact-Citrine* (Riedl *et al.* 2008) flanked by 413 bp and 420 bp of homology upstream of *cxcr4b* exon2, and downstream of the *cxcr4b* stop codon, respectively, was inserted to replace the *cxcr4b* coding sequence in *cxcr4b* exon 2 (amino acid 6–358, the last amino acid before the stop codon) using seamless galK-mediated recombineering (Warming *et al.* 2005). This transgene expresses the first five amino acids from *cxcr4b* exon 1 fused to Lifeact-Citrine from the *cxcr4b* promoter. The final BAC transgene was characterized by *Spe*I and *Eco*RI restriction digestion, sequencing of PCR amplicons of the modified locus, and BAC-end sequencing.

For the *sdf1a:sdf1a-GFP* transgene without the targeting cassette, the BAC clone CH73-199F2 was modified as described above, with the modification that GFP was inserted instead of 3xFlag-4xHA, and that the targeting cassette with the Tol2 sites and the *cryaa:fluorescent protein* transgenesis marker was not inserted into the BAC.

The generation of the *sdf1a:sdf1a-GFP* (with the targeting cassette), *cxcr4b:cxcr4b-Kate2-IRES-GFP-CaaX*, and *cxcr4b:cxcr4b-GFP-IRES-Kate2-CaaX* transgenes was described previously (Lewellis *et al.* 2013; Venkiteswaran *et al.* 2013). Note that the GalK selection cassette will work in the SW102, SW105, and SW106 strains. However, since the SW102 strain is tetracycline resistant, the tetR-based targeting cassettes cannot be used for this strain. BACs from the CHORI-73 BAC library were obtained from the Children’s Hospital Oakland Research Institute (bacpacorders@chori.org), and BACs from the DanioKey BAC library were obtained from ImaGenes GmbH, Germany, (sales@imagenes-bio.de).

### Generation of BAC transgenic lines

BAC transgenes were purified with the nucleobond BAC 100 kit (Clontech), and handled with wide-bore tips to avoid DNA shearing. We coinjected 1 nl of 50–250 ng/μl BAC DNA, and 40 ng/μl *Tol2* mRNA into the lifting cell of the zygote of 0- to 20-min-old embryos. The Tol2 mRNA was transcribed from pCS2FA-transposase (Kwan *et al.* 2007) using the mMessage mMachine SP6 Transcription Kit (Thermo Fisher). However, since BAC DNA is very viscous and difficult to quantify, the BAC DNA concentration and injection volume above are estimations. At 4 days postfertilization (dpf), the rate of mosaic expression of the fluorescent protein in the lens was scored. The BAC DNA amount injected was titrated to yield clutches with 50% or more embryos showing mosaic fluorescent protein expression in the lens. We note that the rate and extent of mosaic fluorescent protein expression in embryos at 4 dpf is dependent on the amount and quality of BAC DNA injected. Clutches with 50% or more embryos showing mosaic fluorescent protein expression in the lens were raised to adulthood. In the case of the *sdf1a:sdf1a-3xFlag-4xHA*; *cryaa:dsRed* BAC transgene injections, embryos with and without mosaic fluorescent protein expression in the lens were raised separately to adulthood. In the case of injections of the *sdf1a:sdf1a-GFP* BAC transgene without the targeting cassette, 20 injected embryos from a clutch were fixed and stained for GFP transcripts *in situ*. Clutches with mosaic GFP mRNA expression in more than 25% of the embryos were raised to adulthood. Stable transgenic larvae were identified by out-crossing adults injected with the transgenes, and by raising larvae positive for the fluorescent transgenesis marker in the lens at 4 dpf. In the case of the *sdf1a:sdf1a-GFP* BAC transgene without the targeting cassette, adult founder fish were identified by staining their offspring for GFP transcripts. The stable line was generated by raising offspring from the founder blindly, and identifying *TgBAC(sdf1a:sdf1a-GFP)* fish by staining their offspring for GFP transcripts. The germline of all potential founder fish (adult fish that were injected as embryos) was screened by analyzing 60 or more of their offspring for fluorescent eyes or GFP mRNA expression unless stable transgenic offspring were identified before reaching this limit. For the *cxcr4b:Lifeact-Citrine* BAC transgene, 63 potential founders were screened; for the *cxcr4b:cxcr4b-Kate2-IRES-GFP-CaaX* BAC transgene, 12 potential founders were screened; for the *sdf1a:sdf1a*-GFP BAC transgene without the targeting cassette, 60 potential founders were screened; for the *sdf1a:sdf1a*-GFP BAC transgene with the targeting cassette, 61 potential founders were screened; for the *sdf1a:sdf1a-3xFlag-4xHA* BAC transgene, 33 potential founders with no fluorescent protein expression in the lens at 4 dpf were screened; and for the *sdf1a:sdf1a-3xFlag-4xHA* BAC transgene, 42 potential founders with fluorescent protein expression in the lens at 4 dpf were screened (for details, see Supporting Information, Table S1, Table S2, Table S3, Table S4, Table S5, and Table S6). The full names of the transgenic lines identified are *TgBAC(sdf1a:sdf1a-GFP)* p1, *TgBAC(sdf1a:sdf1a-GFP*; *cryaa:dsRed)* p1 through p20, *TgBAC(sdf1a:sdf1a-3xFlag-4xHA*; *cryaa:dsRed)* p1 through p16, *TgBAC(cxcr4b:cxcr4b-Kate2-IRES-GFP-CaaX*; *cryaa:dsRed)* p1 through p9, and *TgBAC(cxcr4b:Lifeact-Citrine*; *cryaa:dsRed)* p1 through p16. The lines *TgBAC(sdf1a:sdf1a-GFP*; *cryaa:dsRed)* p10 and *TgBAC(cxcr4b:cxcr4b-Kate2-IRES-GFP-CaaX*; *cryaa:dsRed)* p7 have been described previously (Lewellis *et al.* 2013; Venkiteswaran *et al.* 2013). The lines used for quantification of the *cxcr4b* BAC, *TgBAC(cxcr4b:cxcr4b-GFP-IRES-Kate2-CaaX*; *cryaa:dsRed)* p1, p5, and p7, have also been reported previously (Venkiteswaran *et al.* 2013).

### Quantification of expression levels

Live *cxcr4b:Lifeact-Citrine* and *cxcr4b:cxcr4b-GFP-IRES-Kate2-CaaX* embryos were mounted in 0.5% low-melt agarose/Ringer’s solution (HEPES 5 mM, NaCl 116 mM, KCl 2.9 mM, CaCl_2_ 1.8 mM). Z-stacks were collected with a Leica 40x water dipping lens (NA 0.8), a Leica SP5 II confocal microscope equipped with HyD detectors (Leica Microsystems), and a heated stage (Warner Instruments). The temperature of the water bath was monitored and maintained between 27.9° and 28.4°. All Z-stacks were collected with the HyD detectors in the photon-counting mode, and with identical microscope settings. The laser power was adjusted to 43 μW for the 514 nm laser line, and to 1.00 mW for the 561 nm laser line using a Leica 10x lens (NA 0.3) and an x-cite power meter (Lumen Dynamics Group Inc). Using ImageJ (NIH), a mask was applied to the YFP or RFP channel with a thresholding algorithm to mark the posterior lateral line primordium. All values in the YFP and RFP channels outside of the mask were discarded. All values in the YFP and RFP channels inside of the mask were averaged. The average photon counts per primordium and per embryo were plotted using Prism (GraphPad Software Inc.). A custom ImageJ (NIH) macro language script was written in order to automate this analysis (File S1).

### mRNA and antibody staining

For mRNA staining, RNA probe synthesis and *in situ* hybridization was performed as previously described (Thisse and Thisse 2008). The RNA probe against *sdf1a* was labeled with DIG (Roche), and detected with anti-DIG antibody coupled to alkaline phosphatase (1:5000, Roche) and NBT/BCIP (Roche). For GFP staining, the embryos were fixed for 2 hr at room temperature, dehydrated in 100% methanol, stained with anti-GFP antibody (1:1000, Torrey Pines), and detected with a secondary antibody against rabbit coupled to Cy3 (1:1000, Jackson Immuno Research).

### Image acquisition

Stained *sdf1:sdf1a-GFP* embryos, and live *cxcr4b:Lifeact-Citrine* and *cxcr4b:cxcr4b-GFP-IRES-Kate2-CaaX* embryos, were mounted in agarose as described but on a coverslip with a glass ring and a slide on top to allow for the use of immersion objectives. Embryos were imaged with Leica objectives (10x, NA 0.5, and 40x, NA 1.1) using a Leica SP5 II confocal microscope. Overview images were sum projected using ImageJ, and stitched together using an ImageJ plugin (Preibisch *et al.* 2009).

### Zebrafish strains

Embryos were generated by crossing wild-type Tübingen adults (Haffter *et al.* 1996), and staged as previously described (Kimmel *et al.* 1995).

### Data availability

Fish lines are available upon request. Plasmid sequences and plasmid DNA are available through Addgene (www.addgene.org).

## Results

### A plasmid set encoding BAC targeting cassettes that include Tol2 sites and different transgenesis markers

**Figure 1:**
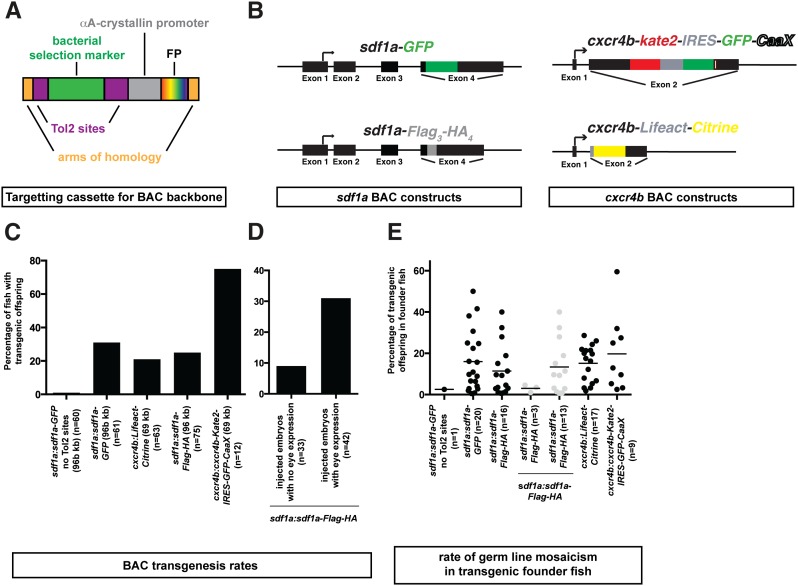
Transgenic constructs and *tol2*-mediated transgenesis rates for BACs. (A) Schematic representation of BAC backbone targeting cassettes containing arms of homology for the BAC backbone (orange), a selection marker (green) flanked by Tol2 sites (purple), followed by the *cryaa* promoter (gray) driving a fluorescent protein (rainbow) in the lens of the eye (see Table 1). (B) Schematic representation of the BAC transgenes. Note that *cxcr4b-GFP-IRES-Kate2-CaaX* is not shown. (C) Rates of transgenesis for the BAC transgenes depicted in B. The size of the transgenes and the number of fish screened (*n*) are indicated. (D) Transgenesis rate of embryos injected with the *sdf1a:sdf1a-Flag_3_-HA_4_* transgene, and presorted for mosaic fluorescent protein expression in the lens compared to injected embryos without mosaic fluorescent protein expression in the lens. The overall rate of transgenesis for the *sdf1a:sdf1a-Flag_3_-HA_4_* transgene is indicated in C. (E) Rate of germline mosaicism in the transgenic founder fish for the transgenes indicated in B. Dots represent the rate of germline mosaicism in individual fish and the horizontal line indicates the median.

To insert *Tol2* *cis*-sequences together with a transgenesis marker into BAC clones, we generated a set of plasmids with targeting cassettes for the backbones of the indigoBAC-based DanioKey BAC library, and the TARBAC-based CHORI-211 and CHORI-73 BAC libraries (http://www.sanger.ac.uk/resources/zebrafish/faq.html). Specifically, we generated plasmid-based PCR templates that contain the two full-length *Tol2* *cis*-sequences (Kawakami *et al.* 2000), the *alphaA-crystallin* (*cryaa*) promoter driving a fluorescent protein (Kurita *et al.* 2003), a bacterial selection marker, and two arms of DNA that are homologous to the BAC backbones. We used Cerulean, Citrine, superfolder GFP (sfGFP), and dsRed as fluorescent markers, and galK flanked by FRT sites, kanaR or tetR as bacterial selection markers. The *cryaa* promoter drives high levels of expression in the lens of larval and adult zebrafish; when driving a fluorescent protein, it serves three purposes. First, it allows for the assessment of the efficiency with which the BAC transgene was injected into embryos based on the proportion of embryos with mosaic fluorescent protein expression in the lens. Second, it is an easily identifiable transformation marker, and third, it is a tool to keep track of adult transgenic fish. Table 1 and Figure 1A contain the names of these plasmids, and the arrangement and orientation of these DNA sequences.

**Table 1:** Names of BAC backbone targeting cassette-containing plasmids

	Plasmid Name	Bacterial Selection Marker	Fluorescent Protein
pTARBAC2	*pBS-TarHom-Tol2-FRT-GalK-cryaa-dsRed*	FRT-galK-FRT	dsRed
	*pBS-TarHom-Tol2-FRT-GalK-cryaa-YFP*	FRT-galK-FRT	Citrine
	*pBS-TarHom-Tol2-FRT-GalK-cryaa-sfGFP*	FRT-galK-FRT	sfGFP
	*pBS-TarHom-Tol2-FRT-GalK-cryaa-CFP*	FRT-galK-FRT	Cerulean
	*pBS-TarHom-Tol2-TetR-cryaa-dsRed*	tetR	dsRed
	*pBS-TarHom-Tol2-TetR-cryaa-YFP*	tetR	Citrine
	*pBS-TarHom-Tol2-kanaR-cryaa-dsRed*	kanaR	dsRed
	*pBS-TarHom-Tol2-kanaR-cryaa-YFP*	kanaR	Citrine
	*pBS-TarHom-Tol2-kanaR-cryaa-sfGFP*	kanaR	sfGFP
	*pBS-TarHom-Tol2-kanaR-cryaa-CFP*	kanaR	Cerulean
pIndigoBAC-536	*pBS-IndHom-Tol2-FRT-GalK-cryaa-dsRed*	FRT-galK-FRT	dsRed
	*pBS-IndHom-Tol2-TetR-cryaa-dsRed*	tetR	dsRed

### The targeting cassettes mediate efficient BAC transgenesis

To test these targeting cassettes, we modified a TARBAC-based and an indigoBAC-based BAC clone. The TARBAC-based BAC clone is 96 kb in size and spans the genomic region of the chemokine *sdf1a*, and the indigoBAC-based BAC clone is 69 kb in size and spans the genomic region of the chemokine receptor *cxcr4b*. Using recombineering (Warming *et al.* 2005), we inserted targeting cassettes into the backbone of these two BACs. The *sdf1a* BAC was modified further to tag Sdf1a on its C-terminus with 3xFlag-4xHA or GFP. The *cxcr4b* BAC was modified further by tagging Cxcr4b on its C-terminus with Kate2-IRES-GFP-CaaX, GFP-IRES-Kate2-CaaX, or by replacing most of the *cxcr4b* coding sequence with *Lifeact-Citrine*, a marker for F-Actin (Riedl *et al.* 2008) (Figure 1B).

**Figure 2:**
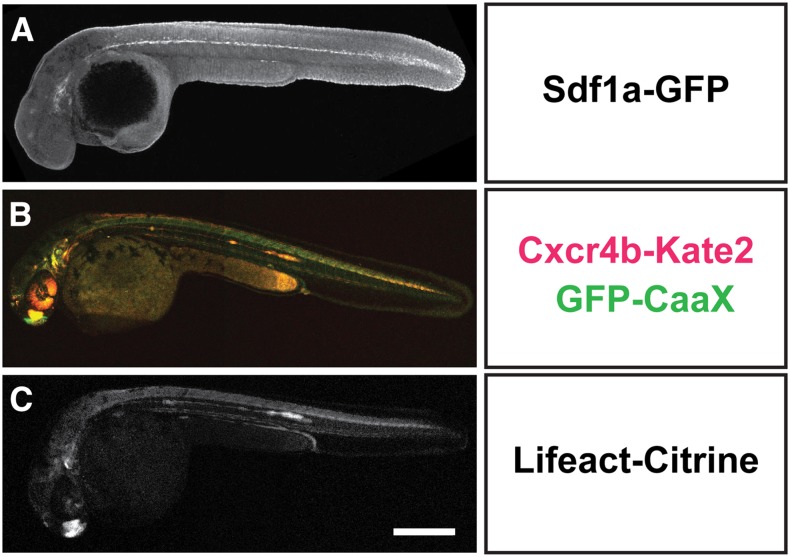
BAC transgenes faithfully recapitulate endogenous expression patterns. Overview images of embryos expressing *sdf1a:sdf1a-GFP*. (A) Stained embryo at 30 hours postfertilization (hpf), (B) *cxcr4b:cxcr4b-Kate2-IRES-GFP-CaaX* (live 36-hpf embryo), and (C) *cxcr4b:Lifeact-Citrine* (live 36-hpf embryo). Images are maximum (A) or sum intensity (B and C) projections of z-stacks. Scale bar corresponds to 300 μm.

To determine the transgenesis rate of these constructs, we microinjected a mixture of the appropriate BAC transgene and mRNA coding for *Tol2* transposase into the lifting cell of the zygote. As a control, we microinjected the 96 kb *sdf1a:sdf1a-GFP* BAC lacking the targeting cassette. The injected embryos were raised to adulthood. We then screened for adult fishes that gave rise to offspring with lenses that express the fluorescent transgenesis marker, or—in the case of the control injections—that express *GFP* mRNA in the locations where *sdf1a* transcripts are expressed. Expression of fluorescent proteins in the lens was scored in live embryos using a fluorescent dissecting scope, and expression of *GFP* mRNA was scored in fixed embryos by *in situ* hybridization. Using this approach, we generated 73 BAC transgenic lines. In total, we generated one *sdf1a:sdf1a-GFP* transgenic line without the *Tol2 targeting cassette*, 20 *sdf1a:sdf1a-GFP* transgenic lines with the *Tol2 targeting cassette*, 16 *sdf1a:sdf1a-3xFlag-4xHA* transgenic lines, 17 *cxcr4b:Lifeact-Citrine* transgenic lines, and nine *cxcr4b:cxcr4b-Kate2-IRES-GFP-CaaX* transgenic lines (Figure 1E, Table S1, Table S2, Table S3, Table S4, Table S5, and Table S6). While the transgenesis rate for the control *sdf1a* BAC lacking the targeting cassette was 1.6% (60 potential founders screened), the transgenesis rate for the 96 kb *sdf1a* BAC carrying the targeting cassette ranged from 21% to 31% (75 and 61 potential founders screened for *sdf1a:sdf1a-3xFlag-4xHA* and *sdf1a:sdf1a-GFP*, respectively) (Figure 1C). The germline mosaicism rate ranged from 0.5% to 50% (Figure 1E, Table S4, Table S5, and Table S6). The transgenesis rate for the 69 kb *cxcr4b* BAC was comparable, ranging from 25% to 75% (63 and 12 potential founders screened for *cxcr4b:Lifeact-Citrine* and *cxcr4b:cxcr4b-Kate2-IRES-GFP-CaaX*, respectively) (Figure 1C), and the mosaicism rate in the germline ranged from 2% to 59% (Figure 1E, Table S1, and Table S2). These transgenesis rates are comparable with previous reports for Tol2-mediated BAC transgenesis using iTol2, for which rates of 10–30% were reported (Suster *et al.* 2011; Bussmann and Schulte-Merker 2011). Although these transgenic lines faithfully recapitulate the endogenous expression pattern (Figure 2), many of them drive very low levels of expression from the *sdf1a* and *cxcr4b* promoter, and would have likely been missed by visual screening for expression. In fact, none of the *sdf1a:sdf1a-GFP* transgenic lines expresses Sdf1a-GFP at detectable levels (Figure 2A). This indicates that the use of a bright and easily identifiable transgenesis marker, such as the *cryaa* promoter driving fluorescent protein expression, in the lens greatly facilitates the isolation of BAC transgenic lines.

### Presorting for injected embryos with mosaic expression in the eye increases the rate of BAC transgenesis

For small, plasmid-based transgenes, one way to increase the rate of transgenesis in zebrafish is to raise only those injected embryos that mosaically express fluorescent protein driven by the promoter of the injected transgene (Rembold *et al.* 2006). To test if this holds true also for larger BAC transgenes, we compared the BAC transgenesis rates of fish that had mosaic fluorescent protein expression in the lens after injection, to fish that had no mosaic fluorescent protein expression in the lens after injection. For the *sdf1a:sdf1a-3xFlag-4xHA* BAC transgene, the transgenesis rate of injected embryos with mosaic fluorescent protein expression in the lens was 31%, compared to 9% for injected embryos without detectable levels of fluorescent protein expression in the lens (42 and 33 potential founders screened for fish with and without mosaic expression of fluorescent protein in the lens, respectively) (Figure 1D). Without presorting, the transgenesis rate for the *sdf1a:sdf1a-3xFlag-4xHA* BAC transgene averages to 21% (Figure 1C). Thus, similar to small transgenes (Rembold *et al.* 2006), presorting embryos for fluorescent protein expression in the lens increases the BAC transgenesis rate significantly.

### BAC transgenic lines provide an estimation of endogenous gene expression levels

**Figure 3:**
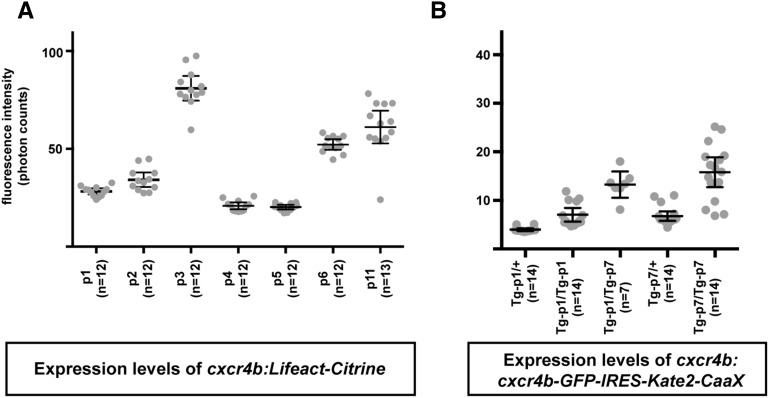
Integration site effects on BAC transgene expression levels. Quantification of the average citrine (A) and Kate2 (B) fluorescence within the posterior lateral line primordium at 36 hpf for the *cxcr4b:Lifeact-Citrine* and *cxcr4b:cxcr4b-GFP-IRES-Kate2-CaaX* transgenes in different transgenic lines. Expression levels vary about three- to four-fold among the different transgenic lines for both BAC transgenes, likely reflecting effects of the integration site on expression levels. Individual measurements of the average fluorescence intensity within the posterior lateral line primordium are indicated by gray dots. Horizontal lines indicate the average fluorescence intensity of the different transgenic lines, and error bars indicate the 95% confidence interval.

It has been suggested that BAC transgenes drive expression at comparable levels independent of their insertion site in the zebrafish genome (Bussmann and Schulte-Merker 2011). To test this idea, we compared the expression levels of the *cxcr4b:cxcr4b-GFP-IRES-Kate2-CaaX* and *cxcr4b:Lifeact-Citrine* transgenes at different insertion sites in the fish genome by quantifying the fluorescence intensity in the posterior lateral line primordium, a tissue that expresses *cxcr4b* (File S1) (Chong *et al.* 2001). We find that the expression levels vary three- to four-fold. However, most BAC transgenic lines drive expression at similar levels (Figure 3, A and B), and transgene copy number correlates linearly with the expression level (Figure 3B). Consistent with observations in mouse (Heintz 2001; Yang and Gong 2005), this suggests that BAC transgenic lines that express at levels lower or higher than the median level of expression represent insertions at sites that promote low or high expression, respectively, while the remaining lines likely reflect the expression levels of the endogenous locus. Additionally, the expression levels of the fluorescent protein driven from the *cryaa* promoter in the lens is generally a good indication of the expression levels of the promoter contained within the genomic DNA fragment of the BAC, but this correlation does not always hold true.

## Discussion

We generated a set of plasmids with BAC targeting cassettes that contain *Tol2* *cis*-sequences and a transgenesis marker, used these targeting cassettes to modify two different BACs, and generated 73 BAC transgenic fish lines by Tol2-mediated transgenesis (Kawakami *et al.* 2000; Kawakami 2007). We find that Tol2-mediated BAC transgenesis with Tol2 sites, and independent selection markers for transgenesis, is an efficient method for inserting large genomic DNA fragments into the zebrafish genome. This approach yields BAC transgenesis rates of more than 20%, which is similar to the rates reported for the iTol2 system (Suster *et al.* 2009a, 2009b, 2011; Bussmann and Schulte-Merker 2011). These large transgenes faithfully recapitulate the endogenous gene expression patterns, and allow for the estimation of the endogenous gene expression levels. Moreover, the inclusion of a transgenesis marker driving the expression of different fluorescent proteins in the lens of the larval and adult eye allows for rapid sorting of transgenic embryos from 2 to 3 dpf onwards and facilitates the maintenance of adult transgenic stocks.

Using classical approaches in zebrafish, transgenic lines are primarily identified based on the fluorescence intensity of fluorescent proteins alone, or fluorescent proteins fused to a protein of interest (Thermes *et al.* 2002; Udvadia and Linney 2003; Kawakami *et al.* 2004; Villefranc *et al.* 2007; Kwan *et al.* 2007; Mosimann *et al.* 2013). Such an approach selects for transgenic lines that drive expression at high levels, but does not easily allow for the identification of transgenes from promoters that drive expression at levels too low to detect by standard fluorescent dissecting microscopes. Consistent with this consideration, we find that the identification of *cxcr4b* BAC transgenic lines based on the expression of the transgenesis marker in the lens yields transgenic lines that express Cxcr4b-GFP or Lifeact-Citrine at levels that are difficult to detect with a standard fluorescent dissecting microscope. More strikingly, none of the *sdf1a* promoter transgenes identified by lens reporter expression drives expression of Sdf1a-GFP at levels that are detectable using a fluorescent dissecting microscope or a laser scanning confocal microscope. These findings suggest that the selection of transgenic lines based on the intensity of fluorescent reporters, or fluorescent fusion proteins expressed from the locus of the gene of interest, may result in failure to identify transgenic lines for genes with weak promoters. Furthermore, this approach could bias toward the selection of transgenic lines that overexpress fluorescent fusion proteins, which would likely confound the interpretation of functional studies.

In summary, this set of targeting cassettes allows for the efficient generation of BAC transgenic lines without an insertion-site bias, the recapitulation of endogenous expression patterns and the estimation of endogenous expression levels, and provide a tool to keep track of adult transgenic fish.

## 

## Supplementary Material

Supporting Information
