# Supplementary material for: A Plasmid Set for Efficient Bacterial Artificial Chromosome (BAC) Transgenesis in Zebrafish
Source: G3 (Bethesda). 2016 Jan 26;6(4):829–34. doi: 10.1534/g3.115.026344 (PMC4825653; doi:10.1534/g3.115.026344)
Supplement: Supporting Information [file supp_6_4_829__index.html]

A Plasmid Set for Efficient Bacterial Artificial Chromosome (BAC) Transgenesis in Zebrafish — Supporting Information 

# A Plasmid Set for Efficient Bacterial Artificial Chromosome (BAC) Transgenesis in Zebrafish

## Supporting Information for Fuentes *et al.*, 2016

**Files in this Data Supplement:**

- Table S1 - Results for the transgenesis rate of the *cxcr4b:Lifeact-Citrine; cryaa:dsRed* transgene. (.pdf, 461 KB)
- Table S2 - Results for the transgenesis rate of the *cxcr4b:cxcr4b-Kate2-IRES-GFP-CaaX; cryaa:dsRed* transgene. (.pdf, 355 KB)
- Table S3 - Results for the transgenesis rate of the *sdf1a:sdf1a-GFP* transgene without *Tol2* cis-sequences. (.pdf, 383 KB)
- Table S4 - Results for the transgenesis rate of the *sdf1a:sdf1a-GFP; cryaa:dsRed* transgene. (.pdf, 377 KB)
- Table S5 - Results for the transgenesis rate of the *sdf1a:sdf1a-3xFlag-4xHA: cryaa:dsRed* transgene of injected fish with no fluorescent protein expression in the lens. (.pdf, 369 KB)
- Table S6 - Results for the transgenesis rate of the *sdf1a:sdf1a-3xFlag-4xHA; cryaa:dsRed* transgene of injected fish with fluorescent protein expression in the lens. (.pdf, 374 KB)
- File S1 - This file contains the ImageJ script that was used to quantify the fluorescent intensity in the primordium shown in Figure 2 (.zip, 2 KB)
