# Supplementary material for: A Plasmid Set for Efficient Bacterial Artificial Chromosome (BAC) Transgenesis in Zebrafish
Source: G3 (Bethesda). 2016 Jan 26;6(4):829–34. doi: 10.1534/g3.115.026344 (PMC4825653; doi:10.1534/g3.115.026344)
Supplement: Supporting Information [file supp_g3.115.026344_TableS1.pdf]

Table S1. Results for the transgenesis rate of the *cxcr4b:Lifeact-Citrine; cryaa:dsRed* transgene.

number of transgenic embryos	number of non-transgenic embryos	total number of embryos	number of screened injected fish	germline mosaicism in %
0	61	61	2	0
0	63	63	2	0
0	63	63	1	0
0	63	63	2	0
0	64	64	2	0
0	66	66	2	0
0	68	68	1	0
0	69	69	2	0
0	72	72	2	0
0	75	75	2	0
0	79	79	2	0
0	88	88	2	0
0	90	90	2	0
0	90	90	1	0
0	94	94	2	0
0	94	94	1	0
0	96	96	2	0
0	96	96	1	0
0	102	102	2	0
0	102	102	1	0
0	109	109	2	0
0	131	131	2	0
0	137	137	1	0
0	145	145	2	0
0	164	164	1	0
0	186	186	2	0
0	200	200	1	0
0	222	222	1	0
1	30	31	1	3.2
2	5	7	1	28.6
3	165	168	1	1.8
4	71	75	1	5.3
4	122	126	1	3.2
6	22	28	1	21.4
8	92	100	1	8.0
17	235	252	1	6.7

19	98	117	1	16.2
21	75	96	1	21.9
24	172	196	1	12.2
25	71	96	1	26.0
25	102	127	1	19.7
27	84	111	1	24.3
31	146	177	1	17.5
33	122	155	1	21.3
34	137	171	1	20
