# Supplementary material for: A Plasmid Set for Efficient Bacterial Artificial Chromosome (BAC) Transgenesis in Zebrafish
Source: G3 (Bethesda). 2016 Jan 26;6(4):829–34. doi: 10.1534/g3.115.026344 (PMC4825653; doi:10.1534/g3.115.026344)
Supplement: Supporting Information [file supp_g3.115.026344_TableS2.pdf]

Table S2. Results for the transgenesis rate of the *cxcr4b:cxcr4b-Kate2-IRES-GFP-CaaX; cryaa:dsRed* transgene.

number of transgenic embryos	number of non-transgenic embryos	total number of embryos	number of screened injected fish	germline mosaicism in %
0	153	153	2	0
0	200	200	1	0
1	18	19	1	5.3
1	29	30	1	3.3
1	39	40	1	2.5
2	6	8	1	25.0
8	17	25	1	32.0
11	29	40	1	27.5
21	142	163	1	12.9
25	17	42	1	59.5
41	388	429	1	9.6
