# Supplementary material for: A Plasmid Set for Efficient Bacterial Artificial Chromosome (BAC) Transgenesis in Zebrafish
Source: G3 (Bethesda). 2016 Jan 26;6(4):829–34. doi: 10.1534/g3.115.026344 (PMC4825653; doi:10.1534/g3.115.026344)
Supplement: Supporting Information [file supp_g3.115.026344_TableS3.pdf]

Table S3. Results for the transgenesis rate of the *sdf1a:sdf1a-GFP* transgene without *Tol2* cis-sequences.

number of transgenic embryos	number of non-transgenic embryos	total number of embryos	number of screened injected fish	germline mosaicism in %
0	100+	100+	1	0
0	100+	100+	1	0
0	100+	100+	1	0
0	100+	100+	1	0
0	100+	100+	1	0
0	100+	100+	1	0
0	100+	100+	1	0
0	100+	100+	1	0
0	100+	100+	1	0
0	100+	100+	1	0
0	100+	100+	1	0
0	100+	100+	1	0
0	100+	100+	1	0
0	100+	100+	1	0
0	100+	100+	1	0
0	100+	100+	1	0
0	100+	100+	1	0
0	100+	100+	1	0
0	100+	100+	1	0
0	100+	100+	1	0
0	100+	100+	1	0
0	100+	100+	1	0
0	100+	100+	1	0
0	100+	100+	1	0
0	100+	100+	1	0
0	100+	100+	1	0
0	100+	100+	1	0
0	100+	100+	1	0
0	100+	100+	1	0
0	100+	100+	1	0
0	100+	100+	1	0
0	100+	100+	1	0
0	100+	100+	1	0
0	100+	100+	1	0
0	100+	100+	1	0

0	100+	100+	1	0
0	100+	100+	1	0
0	100+	100+	1	0
0	100+	100+	1	0
0	100+	100+	1	0
0	100+	100+	1	0
0	100+	100+	1	0
0	100+	100+	1	0
0	100+	100+	1	0
0	100+	100+	1	0
0	100+	100+	1	0
0	100+	100+	1	0
0	100+	100+	1	0
0	100+	100+	1	0
0	100+	100+	1	0
0	100+	100+	1	0
0	100+	100+	1	0
0	100+	100+	1	0
0	100+	100+	1	0
0	100+	100+	1	0
0	100+	100+	1	0
0	100+	100+	2	0
0	100+	100+	1	0
0	100+	100+	1	0
0	83	2	1	2%
