# Supplementary material for: A Plasmid Set for Efficient Bacterial Artificial Chromosome (BAC) Transgenesis in Zebrafish
Source: G3 (Bethesda). 2016 Jan 26;6(4):829–34. doi: 10.1534/g3.115.026344 (PMC4825653; doi:10.1534/g3.115.026344)
Supplement: Supporting Information [file supp_g3.115.026344_TableS4.pdf]

Table S4. Results for the transgenesis rate of the *sdf1a:sdf1a-GFP; cryaa:dsRed* transgene.

number of transgenic embryos	number of non-transgenic embryos	total number of embryos	number of screened injected fish	germline mosaicism in %
0	63	63	1	0
0	65	65	2	0
0	67	67	2	0
0	69	69	1	0
0	71	71	1	0
0	80	80	1	0
0	83	83	2	0
0	89	89	2	0
0	90	90	2	0
0	100	100	1	0
0	100	100	2	0
0	100	100	2	0
0	100	100	2	0
0	103	103	1	0
0	104	104	1	0
0	105	105	1	0
0	108	108	1	0
0	110	110	1	0
0	115	115	2	0
0	120	120	2	0
0	130	130	1	0
0	150	150	2	0
0	152	152	1	0
0	170	170	2	0
0	204	204	2	0
0	204	204	2	0
0	212	212	1	0
1	120	121	1	0.8
2	419	421	1	0.5
3	100	103	1	2.9
3	103	106	1	2.8
4	204	208	1	1.9
5	41	46	1	10.9
10	52	62	1	16.1
10	142	152	1	6.6
10	146	156	1	6.4

11	258	269	1	4.1
14	75	89	1	15.7
15	154	169	1	8.9
16	48	64	1	25.0
18	55	73	1	24.7
19	174	193	1	9.8
51	83	134	1	38.1
65	65	130	1	50.0
99	139	238	1	41.6
150	519	669	1	22.4
183	414	597	1	30.7
