# Supplementary material for: A Plasmid Set for Efficient Bacterial Artificial Chromosome (BAC) Transgenesis in Zebrafish
Source: G3 (Bethesda). 2016 Jan 26;6(4):829–34. doi: 10.1534/g3.115.026344 (PMC4825653; doi:10.1534/g3.115.026344)
Supplement: Supporting Information [file supp_g3.115.026344_TableS5.pdf]

**Table S5.** Results for the transgenesis rate of the *sdf1a:sdf1a-3xFlag-4xHA; cryaa:dsRed* transgene of injected fish with no fluorescent protein expression in the lens.

| number of transgenic embryos | number of non-transgenic embryos | total number of embryos | number of screened injected fish | germline mosaicism in % |
|---|---|---|---|---|
| 0 | 62 | 62 | 1 | 0 |
| 0 | 62 | 62 | 1 | 0 |
| 0 | 64 | 64 | 2 | 0 |
| 0 | 64 | 64 | 2 | 0 |
| 0 | 76 | 76 | 1 | 0 |
| 0 | 76 | 76 | 1 | 0 |
| 0 | 76 | 76 | 1 | 0 |
| 0 | 78 | 78 | 2 | 0 |
| 0 | 87 | 87 | 1 | 0 |
| 0 | 110 | 110 | 2 | 0 |
| 0 | 110 | 110 | 2 | 0 |
| 0 | 110 | 110 | 2 | 0 |
| 0 | 110 | 110 | 2 | 0 |
| 0 | 130 | 130 | 1 | 0 |
| 0 | 160 | 160 | 1 | 0 |
| 0 | 164 | 164 | 1 | 0 |
| 0 | 180 | 180 | 2 | 0 |
| 0 | 180 | 180 | 2 | 0 |
| 0 | 184 | 184 | 1 | 0 |
| 0 | 184 | 184 | 1 | 0 |
| 0 | 200 | 200 | 1 | 0 |
| 1 | 98 | 101 | 1 | 1.0 |
| 2 | 55 | 57 | 1 | 3.5 |
| 3 | 62 | 65 | 1 | 4.6 |
