# Supplementary material for: A Plasmid Set for Efficient Bacterial Artificial Chromosome (BAC) Transgenesis in Zebrafish
Source: G3 (Bethesda). 2016 Jan 26;6(4):829–34. doi: 10.1534/g3.115.026344 (PMC4825653; doi:10.1534/g3.115.026344)
Supplement: Supporting Information [file supp_g3.115.026344_TableS6.pdf]

Table S6. Results for the transgenesis rate of the *sdf1a:sdf1a-3xFlag-4xHA; cryaa:dsRed* transgene of injected fish with fluorescent protein expression in the lens.

number of transgenic embryos	number of non-transgenic embryos	total number of embryos	number of screened injected fish	germline mosaicism in %
0	63	63	2	0
0	63	63	2	0
0	64	64	1	0
0	80	80	1	0
0	80	80	2	0
0	80	80	2	0
0	85	85	1	0
0	100	100	1	0
0	110	110	1	0
0	145	145	2	0
0	146	146	2	0
0	146	146	2	0
0	160	160	2	0
0	160	160	2	0
0	185	185	1	0
0	200	200	2	0
0	200	200	2	0
0	250	250	1	0
1	31	32	1	3.1
1	140	141	1	0.7
2	68	70	1	2.9
3	360	363	1	0.8
4	280	284	1	1.4
7	18	25	1	28.0
8	45	53	1	15.1
9	88	97	1	9.3
16	150	166	1	9.6
20	155	175	1	11.4
22	46	68	1	32.4
23	99	122	1	18.9
40	60	100	1	40.0
